# Notes on *Metaphire multitheca* (Chen, 1938) (Oligochaeta, Megascolecidae) recorded from Vietnam, with descriptions of two new species

**DOI:** 10.3897/zookeys.506.9550

**Published:** 2015-06-01

**Authors:** Anh D. Nguyen, Tung T. Nguyen

**Affiliations:** 1 Institute of Ecology and Biological Resources, Vietnam Academy of Science and Technology, No.18, Hoangquocviet Rd., Hanoi, Vietnam; 2 Department of Biology, School of Education, Cantho University, Cantho City, Vietnam

**Keywords:** Annelida, *Pheretima*, *Amynthas*, earthworm, new species, taxonomy

## Abstract

The paper deals with *Pheretima multitheca multitheca* Chen, 1938 recorded from Vietnam (non *Pheretima multitheca* Chen, 1938 now in *Metaphire* from Hainan Island). As a result, a new species, *Amynthas erroneous* **sp. n.**, is revealed from materials which were previously misidentified as *Pheretima multitheca multitheca*. The new species is obviously distinguished from other *Amynthas* species by multiple spermathecal pores lateroventral in intersegments 5/6/7/8/9, and presence of two pairs of crescentic genital markings in xviii. In addition, another new species, *Amynthas nhonmontis* **sp. n.**, is described and easily recognized by multiple spermathecal pores ventral in intersegments 5/6/7/8 and three pairs of genital markings in xvii, xix and xx.

## Introduction

The species, *Metaphire multitheca* (Chen, 1938) was originally described from Hainan Island (China) in genus *Pheretima* by Chen (1938). The species is recognized by multiple spermathecal pores (more than two spermathecal pores per segment) in vi, vii and viii, two pairs of genital markings in 17/18 and 18/19, and presence of copulatory pouches. The species, was subsequently recorded from various parts of Vietnam by Nguyen (1994), Pham (1995, 2010), Huynh (2005a, 2005b), and Nguyen and Tran (2008). However, all of these authors commented that the population from Vietnam has differences from the original description, such as absence of copulatory pouches, and multiple spermathecal pores in intersegments 5/6/7/8/9.

While examining new materials, we realized that all previous records have been misidentified as *Pheretima multitheca* Chen, 1938 requiring its naming as a new species. In addition, another new species having multiple spermathecae per segment is also described from Vietnam.

## Material and methods

Examined specimens were previously collected from various parts of Vietnam and deposited in:

SORC Soil Organism Research Center;

HNUE Hanoi National University of Education;

CTU Laboratory of Zoology, Cantho University, Cantho City, Vietnam.

Material for DNA barcoding was taken from holotype.

The primer sets, LCO1490 and HCO2198, used in a wide range of invertebrate taxa were used to amplify a fragment of the cytochrome c oxydase subunit I gene (Folmer et al. 1994).

Holotype and paratypes are deposited in the Laboratory of Zoology, Cantho University (= CTU), Cantho City, Vietnam.

Abbreviations: C = Clitellate specimen/specimens, e.g. 5C.

## Taxonomic account

### Family Megascolecidae Rosa, 1891

#### Genus Amynthas

Taxon classificationAnimaliaOpisthoporaMegascolecidae

Kinberg, 1867

Amynthas Kinberg, 1867: 97 et *Amyntas* (laps. praeocc.) pg. 101.
Amyntas (part) – Beddard 1900a: 612.
Nitocris Kinberg, 1867: 102 (praeocc.).
Pheretima – Michaelsen 1900: 234.
Perichaeta (part praeocc.) – Beddard 1895: 388.
Promegascolex Cognetti, 1922 (part see Blakemore et al. 2007)
Pheretima (Pheretima) (part) – Michaelsen 1928: 8; Michaelsen 1934: 15.
Amynthas – Sims and Easton 1972: 211; Blakemore 2002: 149, 2007, 2008.

##### Type species.

*Amynthas aeruginosus* Kinberg, 1867, by monotypy.

##### Distribution.

Widely distributed in the Oriental region, and also found in Australasian and Oceanian regions (Sims and Easton 1972) and distributed worldwide (Blakemore 2002, 2007).

##### Remarks.

Members of the genus can be easily recognized by the presence of intestinal caeca near xxvii, the absence of copulatory pouches, and often by absence of micronephridia on spermathecal ducts. It is noted that about 500+ nominal species have been recorded, but a considerable number are likely synonyms (Blakemore 2002, 2007).

#### Amynthas erroneous sp. n.

Taxon classificationAnimaliaOpisthoporaMegascolecidae

http://zoobank.org/27A9F82B-A620-4EEC-83A7-28B849954C1A

Fig. 1 , Table 1

Pheretima multitheca multitheca – Nguyen 1994: 53; Pham 1995: 68; Huynh 2005a: 89; Huynh 2005b: 20; Nguyen and Tran 2008: 185; Pham 2010: 63.
Non Pheretima multitheca Chen, 1938: 383, fig. 2.

##### Material examined.

*Holotype.* 1C (CTU–EW 071.02–h01) taro garden, Duc Pho commune (108°57'9"E, 14°48'18"N), elevation of 5 m a.s.l., Pho Minh district, Quang Ngai province, Vietnam, 15 April 1995, coll. Huynh Thi Kim Hoi. *Paratypes.* 13C (CTU–EW 071.02–p02) same data as for holotype. *Further material.* 8C (SORC–V.153.01) garden, Duc Pho town, Pho Minh, Quang Ngai, 15 April 1995, coll. Huynh Thi Kim Hoi. Fixed in formalin.

**Figure 1.**
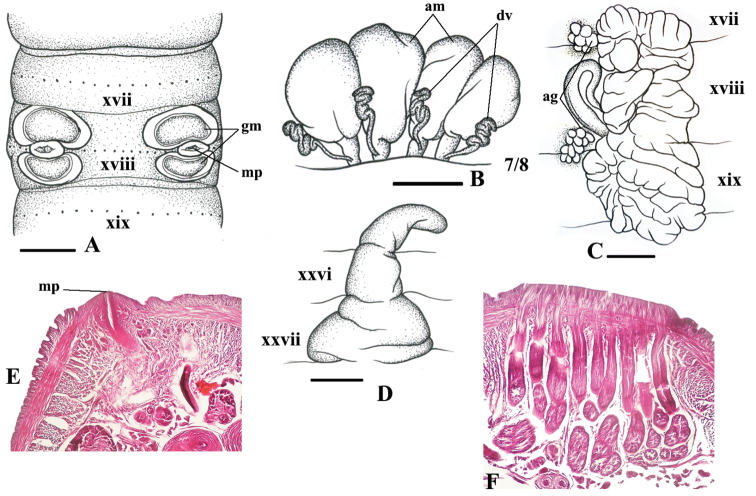
*Amynthas erroneous* sp. n., holotype. **A** Male pore region (mp = male pore; gm = genital markings) **B** Spermathecae, right side on intersegment 7/8 (am = ampulla; dv = diverticulum) **C** Prostate gland (ag = accessory gland) **D** Intestinal caecum **E–F** Transverse body section of segment xviii, male pore (**E**), accessory glands (**F**). Scale bars = 1 mm.

##### Diagnosis.

Medium-size worm. First dorsal pore in 11/12 or 12/13. Prostomium 1/3 epilobous. Multiple spermathecal pores lateroventral in intersegments 5/6/7/8/9. Male pores located in xviii, without copulatory pouches. Two pairs of crescentic genital markings in xviii. Holandric. Testis sacs not separated. Intestinal caeca simple, within xxvii-xxv. Septa 8/9/10 absent.

##### Etymology.

To emphasize the misidentification of the species as *Pheretima multitheca*.

##### Description.

*External characters*: Body cylindrical, medium size. Length 112–150 mm, diameter 3.81–5.0 mm, segments 119–150, weight 1.61–2.94 g. Body coloration uniformly brown. Setae perichaetine, more concentrated ventrally; preclitellar setae sparser than postclitellar setae, 42–46 in v, 37–65 in viii, 61–71 in xxv, 42–72 in xxx, 10–16 between male porophores in xviii; setal distance aa=2ab, zz=1.5zy. First dorsal pore in 12/13, rarely in 11/12. Prostomium ⅓ epilobous. Clitellum annular, xiv-¾xvi, darkish brown, smooth and without setae and dorsal pores. Female pore single, mid-ventral in xiv.

Spermathecal pores round and small, multiple, lateroventral in intersegments 5/6/7/8/9, sometimes invisible. No genital markings in spermathecal region.

Male pores located in porophores in xviii, without copulatory pouches; ventral distance between male porophores about 0.28× body circumference. Two pairs of large, crescentic genital markings in xviii, located in front of and behind male porophores.

*Internal characters*: Septa 6/7/8 thickened, 8/9/10 absent, and 10/11/12/13 relatively thickened. Oesophageal gizzard after 7/8, pear-shaped. Intestinal origin at xv; caeca simple, within xxvii–xxv. Last hearts in xiii. Pharyngeal micronephridia developed in 4/5/6. Lymph glands present from xvii, rarely xvi, and lobulated. Typhlosole simple, lamelliform.

Spemathecae variable, 21–27 altogether in 5/6/7/8/9: 2–4 in 5/6, 4–6 in 6/7, 5–7 in 7/8 and 6–8 in 8/9. Spermathecal ampulla large, oval; duct about ⅓, rarely as much as ½ the length of the ampulla. Diverticula irregularly sinusoidal, folded onto itself several times, enlarged distally, about half length of ampulla; stalk attached to base of duct of ampulla. No accessory glands.

Holandric. Testis sacs not separated, developed in x and xi. Seminal vesicles well developed within xi–xii, yellowish white. Oviduct poorly developed on septum 12/13 posteriorly; a pair of ovaries in xiii. Prostate glands racemose, paired in xvii–xix; prostatic ducts C-shaped. Two accessory glands present.

##### DNA.

COI barcode data not yet available.

##### Habitat and ecology.

The species was found in soils in which old growth trees had been grown. No other ecological data had been recorded.

##### Distribution.

Previous misidentifications of *Pheretima multitheca multitheca* were from Quang Tri (Quang Tri town); Thua Thien Hue (Huong Tra; Hue; Nam Dong; Phu Loc); Danang; Quang Nam (Que Son); Quang Ngai (Quang Ngai city; Duc Pho); Binh Dinh; Dak Nong (Ta Dung Mts.) (Nguyen 1994, Pham 1995, 2010, Huynh 2005a, 2005b, Nguyen and Tran 2008).

##### Remarks.

This new species was previously misidentified as *Pheretima multitheca multitheca* Chen, 1938 (= *Metaphire multitheca*), which was originally known only from Hainan Island. Both species share multiple spermathecal pores per segment and presence of intestinal caeca. However, *Amynthas erroneous* sp. n. has multiple spermathecal pores in intersegments 5/6/7/8/9, and lacks copulatory pouches while *Metaphire multitheca* (Chen, 1938) has multiple spermathecal pores located behind setal rings of segments vi, vii, viii, and presence of copulatory pouches. The new species is also fairly similar to *Metaphire multitheca dipapillata* (Thai & Tran, 1986), now *Metaphire dipapillata* stat. n.; both species have multiple spermathecal pores in intersegments 5/6/7/8/9, and genital markings associated with the male pores. However, *Amynthas erroneous* sp. n. lacks copulatory pouches and has two pairs of crescentic genital markings in xviii, whereas *Metaphire dipapillata* has copulatory pouches and only a pair of genital markings in 17/18, located in front of male porophores. Marker characters of three species, *Amynthas erroneous*, *Metaphire dipapillata* and *Metaphire multitheca* are presented in Table 1.

**Table 1.** Marker characters of three species, *Amynthas erroneous* sp. n., *Metaphire dipapillata* (Thai & Tran, 1986), stat. n., and *Metaphire multitheca* (Chen, 1938).

No	Characters	*Amynthas erroneous*	*Metaphire dipapillata*	*Metaphire multitheca*
1	Length (mm)	112–150 mm	115–180 mm	155 mm
2	Diameter (mm)	3.81–5.0 mm	5–7 mm	7 mm
3	Weight	1.61–2.94 g	4.2–7.0 g	?
4	Segments	119–150	103–124	95
5	Setae between male porophores	10–16	10–11	4
6	Coloration	uniformly brown	Dorsa whitish grey, ventra paler	Dorsa darkish grey, ventra paler
	Clitellum	xiv–¾xvi	xiv–xvi	xiv–xvi
7	Prostomium	⅓ epilobous	epilobous	⅓ epilobous
8	First dorsal pore	12/13, rarely 11/12	11/12	12/13
9	Spermathecal pores	Multiple in 5/6/7/8/9	Multiple in 5/6/7/8/9	Multiple in vi, vii, viii
10	GM near spermathecae	Absent	Absent	Absent
11	GM in male region	Two pairs in xviii	A pair in 17/18	Two pairs in xviii
12	Copulatory pouches	Absent	Present	Present
13	Spermathecae	21–27	30–40	30–32
14	Male sexual system	Holandric	Holandric	Holandric
15	Pharyngeal micronephridia	4/5/6	5/6/7/8	5/6/7
16	Septa 8/9/10	Absent	Absent	Absent

The new species is also distinguished from other 5/6/7/8/9 polythecate species such as *Polypheretima bifaria* (Michaelsen, 1924) from New Guinea, *Polypheretima polytheca* (Beddard, 1900b) from Malay penisula, *Polypheretima koyana* Michaelsen, 1934 from Sarawak, and *Metapheretima elrondi* Easton, 1979 from New Guinea by having intestinal caeca. Althought it shares with *Amynthas bleckwenni* (Ude, 1925) from Borneo the multiple spermathecae at 5/6/7/8/9, and having intestinal caeca, the new species differs from *Amynthas bleckwenni* in having two pairs of crescentic genital markings in front of and behind male pores on xviii.

We herein raised the subspecies *Metaphire multitheca dipapillata* to full rank as *Metaphire dipapillata* stat. n., and also restore *Metaphire multitheca multitheca* as *Metaphire multitheca*.

The new species is widely distributed in central parts and highlands of Vietnam. Surprisingly, it has never yet been found in northern and southern Vietnam.

#### Amynthas nhonmontis sp. n.

Taxon classificationAnimaliaOpisthoporaMegascolecidae

http://zoobank.org/6231C5E9-8812-419D-BDB9-34007C10B1F4

Fig. 2

##### Material examined.

*Holotype.* 1C (CTU.EW 023–h01) natural forest, Nhon mountain, (104°56'09.2"E, 10°35'39.6"N), elevation of 56 m a.s.l., Tinh Bien district, An Giang province, Vietnam, 7 November 2010, coll. Nguyen Thanh Tung. *Paratypes.* 6C (CTU. EW023–p02) same data as for holotype.

**Figure 2.**
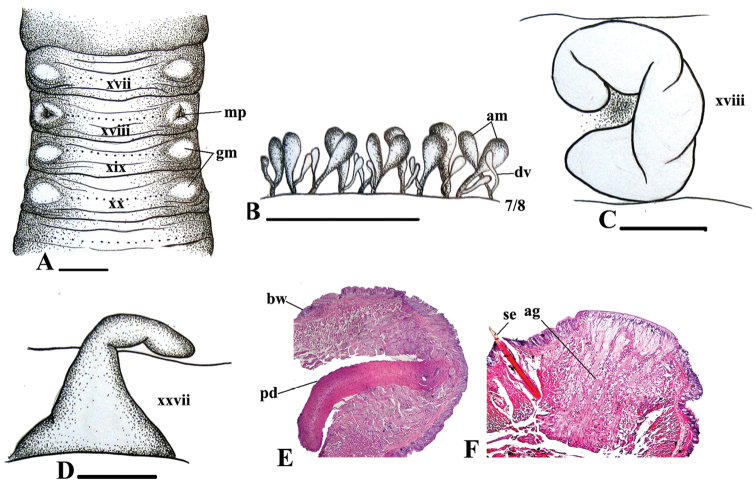
*Amynthas nhonmontis* sp. n., holotype. **A** Male region (mp = male pore; gm = genital markings) **B** Spermathecae, right side on intersegment 7/8 (dv = diverticulum, am = ampulla) **C** Prostate **D** Intestinal caecum **E** Transverse body section of male porophore in xviii (bw = body wall, pd = prostatic duct) **F** Transverse body section of genital markings in xvii (ag = accessory gland). Scale bars = 1 mm.

##### Diagnosis.

Medium-size worm. First dorsal pore in 10/11. Prostomium prolobous. Multiple spermathecal pores ventral in intersegments 5/6/7/8. Male pores in xviii, without copulatory pouches. Three pairs of genital markings present in xvii, xix and xx, rarely more or less than three pairs. Holandric. Intestinal caeca simple, from xxvii. Septa 8/9/10 absent, 10/11 thickened.

##### Etymology.

“*nhonmontis*”, after locality.

##### Description.

*External characters*: Body cylindrical, medium size. Length 103–106 mm, diameter 3.28–4.05 mm, segments 138–168, weight 0.91–1.65 g. Living specimens whitish pink while preserved specimens uniformly whitish brown; clitellum darkish brown.

Prostomium prolobous. First dorsal pore in 10/11. Setae perichaetine; preclitellar setae stouter and thicker than postclitellar setae, 42–51 in viii, 31–36 in xxx, 5–8 between male porophores in xviii; setal distance aa=ab, zz=zy. Clitellum annular, xiv–xvi, without setae and dorsal pores. Female pore single, mid-ventral in xiv. Body wall thinned, especially segments after male region having very thin wall. Preclitellar segments obviously shorter than postclitellar ones.

Multiple spermathecal pores ventral in intersegments 5/6/7/8. No genital markings in spermathecal region. Male porophores flattened; male pores located in setal ring in xviii, without copulatory pouches. Ventral distance between male porophores about 0.3x body circumference. Genital markings usually three pairs in setal rings in xvii, xix and xx, rarely more or less than three pairs; genital markings arranged in longitudinal line with male pores.

*Internal characters*: Septa 5/6/7/8 and 10/11 thickened, 8/9/10 absent, 11/12/13 very thin. Oesophageal gizzard within viii–x. Intestinal origin at xv; caeca small, simple within xxvii–xxvi or xxvii–½xxv. Last hearts in xiii. Pharyngeal micronephridia well-developed on septa 4/5/6. Lymph glands lobulated from xxvii. Typhlosole simple, lamelliform.

Spermathecae small, about 43–48 altogether in intersegments 5/6/7/8: 11–13 in 5/6, 15–17 in 6/7, and 17–18 in 7/8. Spermathecal ampulla very small, clavate, yellowish brown; duct extremely short. Diverticulum shorter and attached directly to duct of ampulla. No accessory glands.

Holandric. Testis sacs separated. Seminal vesicles poorly developed in xi–xii. Oviduct on septum 12/13 ventrally. Ovaries minute (not clearly found). Prostate glands poorly developed, largely lobulated, only within xviii; prostatic ducts undetected, covered body wall. Accessory glands concealed within body walls.

##### DNA.

COI barcode data (partial) is for holotype uploaded to GenBank with accession number KR676559.

##### Habitats and ecology.

This species has been found only in Nhon mountain (Tinh Bien district) in An Giang province. Adult specimens were only collected during the rainy season (from October to March) in southern Vietnam and found in heavy clays at the foothill of Nhon mountain.

##### Remarks.

*Amynthas nhonmontis* sp. n. differs from other *Amynthas* species in having multiple spermathecal pores ventral in intersegments 5/6/7/8 and three pairs of genital markings in xvii, xix and xx. The new species is superficially similar to *Polypheretima elongata* (Perrier, 1872) due to external morphology: body cylindrical, multiple spermathecae per segment, presence and arrangement of genital markings near male pores. However, it is clearly distinguished from *Polypheretima elongata* by presence of intestinal caeca, three spermathecal segments, absence of copulatory pouches.

Its DNA barcode is definitive for the new species (nearest BLAST result is *Amynthas morrisi* at 87%).

## Conclusion

Two new species of the genus *Amynthas* Kinberg, 1867 are described. Both of them are characterised by multiple spermathecal pores per segment, lack of copulatory pouches, and presence of intestinal caeca from xxvii. To date, only eight species having multiple (more than two) spermathecae per segment have been found in Vietnam. They are arranged in three genera: *Polypheretima* Michaelsen, 1934, *Amynthas* Kinberg, 1867 and *Metaphire* Sims & Easton, 1972, namely:

*Polypheretima spiridonovi* (Thai, 1996), from Khanh Hoa province, southern Vietnam (stat. n. from Blakemore 2007, 2008).

*Polypheretima mekongmontis* Nguyen, Tran & Nguyen, 2014, from Kien Giang province, southern Vietnam.

*Polypheretima cattienensis* Nguyen, Tran & Nguyen, 2015, from Dong Nai province, southern Vietnam.

*Polypheretima militium* Nguyen, Tran & Nguyen, 2015, from Dong Nai province, southern Vietnam.

*Polypheretima cordata* Nguyen, Tran & Nguyen, 2015, from Dong Nai province, southern Vietnam.

*Amynthas erroneous* Nguyen, Tran & Nguyen, sp. n., from central and highlands.

*Amynthas nhonmontis* Nguyen, Tran & Nguyen, sp. n., from An Giang province, southern Vietnam.

*Metaphire dipapillata* (Thai & Tran, 1986), stat. n. from Nghe An province, central Vietnam.

## Supplementary Material

XML Treatment for Amynthas

XML Treatment for Amynthas erroneous

XML Treatment for Amynthas nhonmontis
